# Low genetic diversity and shallow population structure in the endangered vulture, *Gyps coprotheres*

**DOI:** 10.1038/s41598-019-41755-4

**Published:** 2019-04-02

**Authors:** Courtneë Kleinhans, Sandi Willows-Munro

**Affiliations:** School of Life Sciences, University of KwaZulu-Natal, Pietermaritzburg, South Africa

**Keywords:** Genetic variation, Behavioural genetics

## Abstract

Globally, vulture species are experiencing major population declines. The southern African Cape vulture (*Gyps coprotheres*) has undergone severe population collapse which has led to a listing of Endangered by the IUCN. Here, a comprehensive genetic survey of *G. coprotheres* is conducted using microsatellite markers. Analyses revealed an overall reduction in heterozygosity compared to other vulture species that occur in South Africa (*Gypaetus barbatus*, *Necrosyrtes monachus*, and *Gyps africanus*). Bayesian clustering analysis and principal coordinate analysis identified shallow, subtle population structuring across South Africa. This provides some support for regional natal philopatry in this species. Despite recent reductions in population size, a genetic bottleneck was not detected by the genetic data. The *G. coprotheres*, however, did show a significant deficiency of overall heterozygosity. This, coupled with the elevated levels of inbreeding and reduced effective population size, suggests that *G. coprotheres* is genetically depauperate. Given that genetic variation is considered a prerequisite for adaptation and population health, the low genetic diversity within *G. coprotheres* populations is of concern and has implications for the future management and conservation of this species.

## Introduction

Examining the spatial distribution of genetic diversity, within and among populations, is important in the management and conservation of threatened taxa^1,2^. Habitat fragmentation and reduced effective population size can affect the genetic structure of endangered species^3^, by increasing levels of inbreeding and genetic drift within populations which in turn reduces the amount of genetic diversity within the gene pool^4,5^. Reduced genetic diversity in turn can have detrimental effects on species ability to recover from demographic, environmental and genetic stochasticity^6^, and can be a contributing factor in reduced long- and short-term survival of endangered species^3,7^.

Global vulture populations have plummeted in recent years^7–10^. During the last decade, Africa and Asia have experienced the highest decrease in vulture populations^8,9,11,12^, as such these species are conservation priorities. There are 23 extant vulture species worldwide, of which 16 species are currently classified as threatened to critically endangered^10^. As a result, the majority of species are now endangered or critically endangered^10^. African vultures are particularly vulnerable, with a recent study suggesting that populations of six of the eleven African species have declined by an average of 62% over the past 30 years^9^. Despite this alarming trend, little is known about the population genetics of African vultures and what impact the reduction in population size has had on the genetic diversity of these birds. Conducting thorough genetic assessments of wide-ranging, highly mobile species, such as vultures, which move between countries or even between continents, poses a considerable challenge. For vultures, and many other raptors, direct estimates of abundance, natal philopatry and dispersal are particularly challenging because individuals are not only difficult to capture and mark, but re-capture rates are also very low^13–15^. Genetic methods can be valuable tools to evaluate genetic diversity, effective population size and gene flow among fragmented populations and have shown to be useful in highly mobile species^15–17^.

Although the Cape vulture (*Gyps coprotheres*) is one of the most well studied vulture species in southern Africa, after decades of conservation efforts the population continues to decline^9,12,18,19^. The species is currently listed as Endangered on the International Union of Conservation of Nature (IUCN) red data list^19^. The decline of this species is largely attributed to anthropogenic factors including habitat loss, reduced food supply and poisoning^20^. Current estimates suggest a wild population of approximately 9400 individuals^21^.

**Figure 1:**
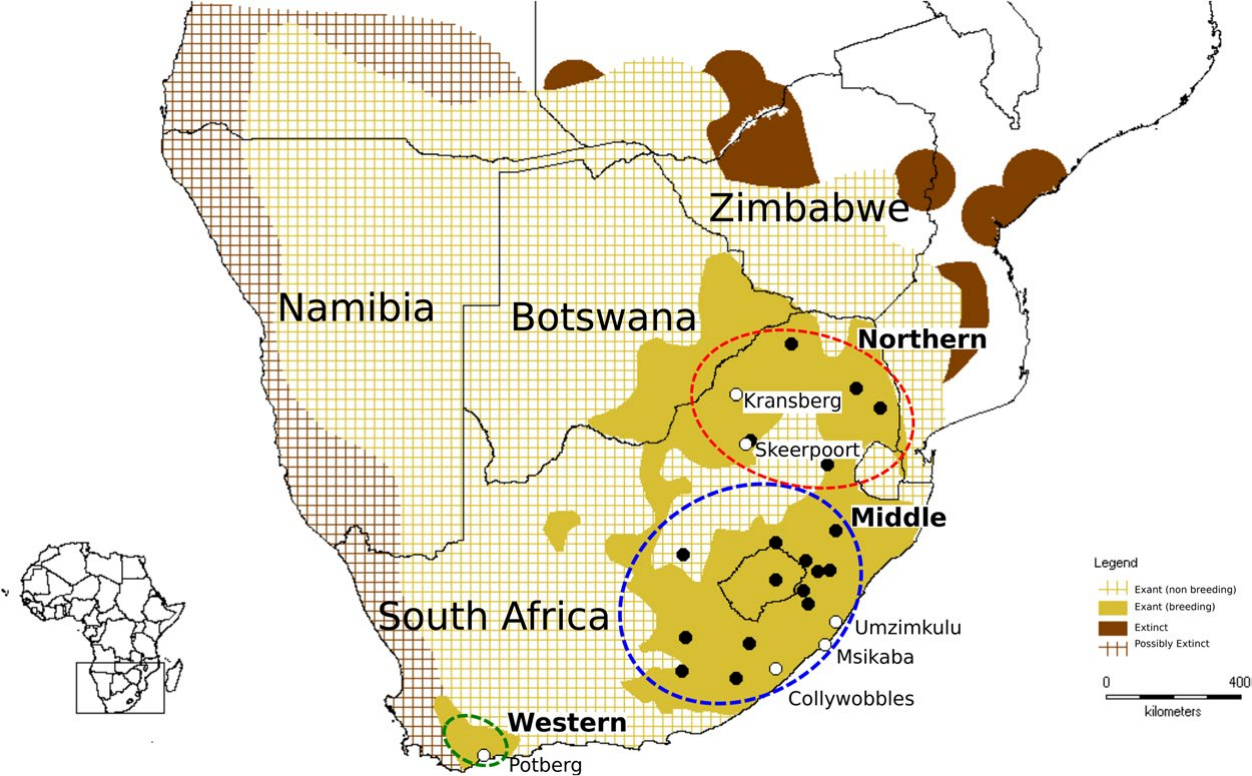
Distribution of *Gyps coprotheres*. Extant and extinct geographical distribution of *Gyps coprotheres* is shown. The 24 sampling localities for the 605 *Gyps coprotheres* included in the present study are shown (black dots). White dots indicate the six breeding colonies. Northern, Middle and Western regional grouping are also shown.

The distribution of the species has also undergone a significant reduction with birds no longer breeding in much of their historical range (Fig. 1). This species flies extensive distances to forage, and has been sighted in Angola, Namibia, Botswana, Zimbabwe, Mozambique, South Africa, Lesotho and Swaziland, but breeding colonies are almost exclusively restricted to South Africa^19,22^. The home ranges for juvenile birds are much larger than that of breeding adults covering a foraging range up to 482 276 km^2^ ^13^. Younger birds are inefficient feeders and forage more widely to avoid feeding competition with adults^22–25^. Literature indicates that individuals return to their natal colony to settle and breed^26^, but natal philopatry has only been confirmed in a few ringing studies^27,28^. There are currently three core *G. coprotheres* breeding populations in South Africa: one in northern South Africa (Limpopo and Mpumalanga provinces); the second in the high lying regions of the KwaZulu-Natal and Eastern Cape provinces of South Africa and Lesotho^14,18,29^; and an small isolated breeding colony in the Western Cape province of South Africa^18^. The connectivity of these regional populations is unknown. Strong natal philopatry should result in pronounced populations substructure even in species such as vultures that travel extensive distances.

This study aims to determine how the recent dramatic reduction in population size has shaped the genetic diversity of *G. coprotheres*. The genetic diversity of South African *G. coprotheres* populations was evaluated using microsatellite loci. The genetic diversity was compared to three ecologically similar vulture species also found in South Africa, but which have wider continental-scale distributions, namely the near-threatened bearded vulture (*Gypaetus barbatus*); the critically endangered hooded vulture (*Necrosyrtes monachus*); and the critically endangered white-backed vulture (*Gyps africanus*)^10^. In addition, this study aims to examine the regional connectivity among six breeding colonies and provide insight into natal philopatry in *G. coprotheres*.

## Results

Thirteen microsatellite loci were amplified in 605 *G. coprotheres* individuals collected from across the southern African distribution of the species. The missing data included in the final data set varied across loci, but was minimal (mean = 11%). Identity analysis estimates showed that all specimens were unique (i.e. no identical specimens) and all 605 *G. coprotheres* individuals were used in subsequent analyses. The mean null allele (No) frequency across the 13 microsatellite loci for *G. coprotheres* data was 5.4% (Supplementary Table 1). The paired t-tests did not indicate significant difference (p-value > 0.05) between uncorrected and correct F_ST_ values, suggesting that null alleles have a very limited effect on the genetic structuring analyses in South African *G. coprotheres*. Therefore all 13 microsatellite loci were used in subsequent analyses.

### Genetic diversity

Genetic diversity estimates indicated that all 13 loci were polymorphic (Table 1). No signs of linkage disequilibrium were detected in the data. Six loci (BV11, BV12, BV13, Gf3H3, Gf9C and Gf11A4) were identified as moderately to highly informative (PIC > 0.5). The number of alleles per locus ranged from 6 (BV2) to 21 (Gf9C). All loci deviated significantly from Hardy-Weinberg equilibrium except for Gf11A4 (HWD >0.004, Table 1). One locus showed elevated levels of heterozygosity (BV11, F = −0.38). Seven loci (BV5, BV6, BV9, BV13, BV14, BV20 and Gf8G) showed signs of heterozygote deficiency (F > 0.15). The overall genetic diversity varied among the 24 sampling localities across South Africa (Supplementary Table 1). The mean number of alleles ranged from 0.54 (Mala Mala) to 7.39 (Highmoor). The observed heterozygosity ranged from 0.08 (Mala Mala) to 0.47 (Smithfield). The inbreeding coefficient ranged from −1.00 (Mala Mala) to 0.29 (Thomas River).

**Table 1:** Summary statistics of the 605 *Gyps coprothere*s genotyped in the present study.

Locus	A	No	F_ST_^A^	F_ST_^B^	Ho	uHe	HWD	F	PIC
BV2	6	0.01	0.05	0.06	0.17	0.17	0.00	0.00	0.16
BV5	8	0.06	0.11	0.19	0.02	0.05	0.00	0.49	0.05
BV6	13	0.09	0.21	0.24	0.05	0.11	0.00	0.50	0.11
BV9	9	0.05	0.04	0.09	0.02	0.04	0.00	0.39	0.04
BV11	15	0.01	0.01	0.01	0.83	0.60	0.00	−0.38	0.54
BV12	20	0.02	0.01	0.01	0.79	0.84	0.00	0.05	0.82
BV13	10	0.20	0.01	0.02	0.26	0.57	0.00	0.54	0.50
BV14	11	0.05	0.04	0.06	0.12	0.16	0.00	0.21	0.15
BV20	7	0.06	0.01	0.02	0.16	0.19	0.00	0.16	0.18
Gf3H3	14	0.02	0.01	0.02	0.62	0.59	0.00	−0.06	0.55
Gf8G	15	0.10	0.03	0.04	0.26	0.35	0.00	0.25	0.32
Gf9C	21	0.02	0.01	0.01	0.85	0.85	0.00	0.01	0.84
Gf11A4	10	0.01	0.00	0.00	0.74	0.75	0.04	0.01	0.70
Mean	12	0.05	0.04	0.06	0.38	0.40	0.00	0.17	—

Number of alleles (A), null allele frequency (No), uncorrected and corrected fixation indices (F_ST_), observed heterozygosity (Ho), unbiased expected heterozygosity (uHe), deviation from Hardy-Weinberg (HWD) p-value, inbreeding coefficient (F) and polymorphic information content (PIC). Null allele frequency was estimated using the EM algorithm. The F_ST_^A^ uncorrected and F_ST_^B^ null allele corrected.

Based on collection locality samples were grouped into three geographic regions (Western, Middle and Northern regions see Fig. 1). Genetic diversity estimates varied among the three geographic regions (Table 2). The number of alleles were the lowest in the Western region (45 alleles) and highest in the Middle region (134 alleles). Although the mean number of alleles differed among the three geographic regions, with the Middle region having the highest mean number of alleles (Ā = 10.308; SE = 1.082), when allelic richness was estimated using the rarefaction index (accounting for the differences in sample sizes among the regions) little difference in allelic richness was observed. The number of private alleles ranged from 0.41 alleles (Western) to 0.79 alleles (Northern). Similar levels of observed heterozygosity were observed across the three regions (observed heterozygosity <0.39). No signs of excess heterozygosity were observed in any of the three regions. The Middle region and the Northern region showed signs of heterozygote deficiency (F > 0.15).

**Table 2:** Genetic diversity estimates for the 605 *Gyps coprotheres* grouped by geographic region and for the 266 *Gyps coprotheres* individuals grouped by breeding colony.

		N	A	Ā	A_R_	A_P_	Ho	uHe	F
Region N = 605	Western	18	45	3.46 (0.75)	3.36	0.41	0.33 (0.10)	0.34 (0.09)	0.06 (0.08)
	Middle	462	134	10.31 (1.08)	3.96	0.74	0.38 (0.10)	0.40 (0.09)	0.15 (0.07)
	Northern	125	95	7.31 (1.09)	4.14	0.79	0.38 (0.09)	0.43 (0.08)	0.19 (0.09)
	Mean		91	7.03 (0.72)	3.82	0.64	0.37 (0.05)	0.39 (0.05)	0.14 (0.05)
Colony N = 266	Potberg	18	45	3.46 (0.75)	3.36	0.4	0.33 (0.10)	0.34 (0.09)	0.06 (0.08)
	Collywobbles	66	57	4.39 (0.82)	3.39	0.20	0.37 (0.10)	0.35 (0.09)	−0.05 (0.07)
	Msikaba	50	58	4.46 (0.95)	3.65	0.21	0.38 (0.10)	0.39 (0.09)	0.06 (0.09)
	Umzimkulu	53	54	4.15 (0.93)	3.51	0.17	0.38 (0.10)	0.38 (0.09)	−0.01 (0.06)
	Skeerpoort	43	52	4.00 (0.89)	3.43	0.19	0.35 (0.09)	0.39 (0.09)	0.06(0.10)
	Kransberg	36	50	3.85 (0.75)	3.44	0.08	0.38 (0.09)	0.39 (0.08)	0.06 (0.08)
	Mean	44	53	4.05(0.34)	3.46	0.21	0.37 (0.04)	0.38 (0.04)	0.03 (0.03)

Number of individuals (N), total number of alleles (A), mean number of alleles (Ā), allelic richness (A_R_), private alleles (A_P_), observed heterozygosity (Ho), unbiased expected heterozygosity (uHe), and inbreeding coefficient (F). Allelic richness for each region was based on the minimum number of gene copies. Standard errors are shown in parentheses.

In addition to the regional grouping, 266 samples collected from six breeding colonies were analysed separately. Genetic diversity also varied among the six breeding colonies (Table 2). The number of alleles ranged from 45 alleles in Potberg to 58 alleles in Msikaba. Allelic richness estimates showed little difference across the six colonies. Similar levels of heterozygosity were observed in all six colonies (observed heterozygosity <0.39). No signs of excess heterozygosity were observed in any of the six colonies. The inbreeding coefficient ranged from −0.045 in Collywobbles to 0.062 in Potberg.

South African representatives of *G. barbatus* (n = 54), *N. monachus* (n = 54), and *G. africanus* (n = 68) were also included and genotyped using the same microsatellite loci, to allow for direct comparison of genetic diversity values. The mean observed heterozygosity in *G. coprotheres* (Ho = 0.38) was much lower than that observed in *G. barbatus* (Ho = 0.50), *N. monachus* (Ho = 0.71), and *G. africanus* (Ho = 0.65). *Gyps coprotheres* and the much smaller, isolated South African *G. barbatus* population showed elevated levels of inbreeding (mean F = 0.17). Inbreeding was not detected in the other two vulture species *N. monachus* F = −0.07 and *G. africanus* F = 0.07 (Supplementary Table 2).

### Population structure

**Figure 2:**
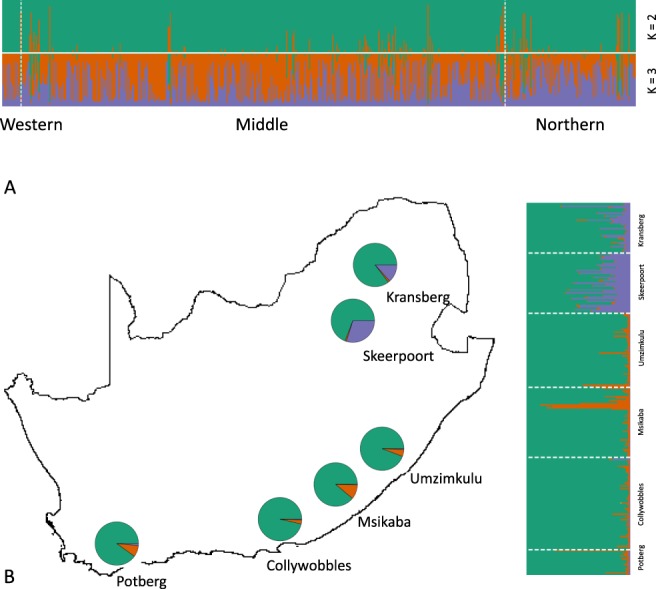
STRUCTURE bar plots the 605 *Gyps coprotheres* grouped by geographic region (**A**) and 266 individuals collected at the six breeding colonies (**B**). Each vertical line in the bar plot represents an individual and is coloured according to individual’s estimated membership coefficient (Q) values. Distribution of mean Bayesian assignment probabilities for each grouping is shown on the maps.

Two optimal genetic clusters were recovered from the Bayesian clustering analysis conducted on the 605 *G. coprotheses*, including all 24 sampling localities (K = 2, delta K = 42.16; Supplementary Table 3). Figure 2 shows the structure barplots for all 605 *G. coprotheses* samples grouped by geographic region for K = 2 and K = 3. No distinct geographic structure was observed in the data across both K-values. However, significant genetic differentiation was observed in all region pairs shown in the pairwise F_ST_ values reported in Supplementary Table 4.

The second Bayesian clustering analysis, conducted using only the 266 *G. coprotheses* collected at the six breeding colonies, recovered three genetic clusters (K = 3, delta K = 11.05; Fig. 2, Supplementary Table 3). All three genetic clusters were again present across the six breeding colonies. In this case, the two colonies (Skeerpoort and Kransberg) are distinct from other colonies. Surprisingly, the geographically isolated Potberg colony in Western Cape, South Africa is not distinguishable from colonies in Eastern Cape and Kwazulu-Natal provinces. However, significant pairwise F_ST_ values (p-value < 0.003) were recovered between the Potberg colony and all colony pairs and the Skeerpoort colony and all colony pairs as shown by pairwise F_ST_ estimates reported in Supplementary Table 5. Pairwise F_ST_ analysis showed no clear genetic partitioning when individuals were assigned to localities (Supplementary Table 6). Mantel tests showed no correlation between pairwise geographic distances and pairwise genetic distances (individuals grouped by locality: R = 0.011, p-value = 0.317; individuals grouped by colony: R = 0.052, p-value = 0.105).

**Figure 3:**
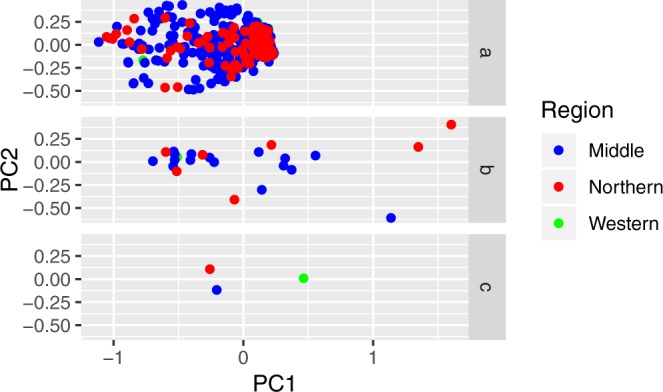
Principle coordinate analyses for the 605 *Gyps coprotheres*. Graph a shows all 605* G. coprotheres* samples. Graph b shows analysis for all samples grouped into the 24 sampling localities. Graph c shows analysis of all samples grouped by geographic region. The colours represent the regional groupings where Western is green, Middle is blue and Northern is red.

Principle coordinate analysis (PCoA) was performed on *G. coprotheses* individuals and *G. coprotheses* samples grouped by populations (where population is either locality, region or colony groupings). Figure 3 shows the PCoA for all 605 *G. coprotheres* sampled. When all 605 *G. coprotheres* individuals were analysed (Fig. 3 graph a) no correlation between individual genetic distance and locality was observed. Similarly, no correlation was observed when samples were grouped by the 24 sampling localities (Fig. 3 graph b). In contrast, when individuals were grouped by the three geographic regions (Fig. 3 graph c), all three regions were genetically distinguishable.

**Figure 4:**
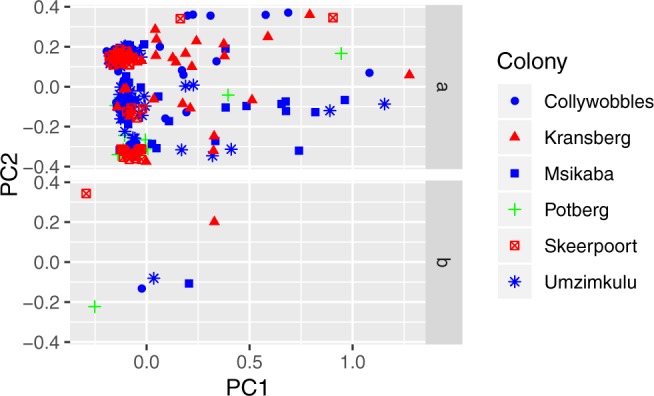
Principle coordinate analyses for the 266 *Gyps coprotheres* collected from six breeding colonies across South Africa. Graph a shows all 266 individuals sampled from the six breeding colonies. Graph b shows samples grouped into the six breeding colonies. The colours represent the regional groupings where Western is green, Middle is blue and Northern is red.

In PCoAs for the 266 *G. coprotheres* individuals collected at the six breeding colonies (Fig. 4) no correlation between individual genetic distances and colonies were observed (Fig. 4 graph a). The PCoA showing individuals grouped by colony (Fig. 4 graph b), support the STRUCTURE results by grouping together the three colonies from the Eastern Cape and Kwazulu-Natal provinces (Collywobbles, Msikaba and Umzimkulu). Interestingly, the PCoA was able to separate the Skeerpoort and Kransberg colonies.

AMOVA results reported that the global F_ST_ values for *G. coprotheses* individuals grouped by sampling locality, *G. coprotheses* assigned to geographic regions and only *G. coprotheses* collected at the six breeding colonies deviated significantly from zero (F_ST_ > 0.01; p-value = 0.00). The majority of genetic variation, however, occurred within individuals (64–77%; Supplementary Table 7).

### Population connectivity

Migration rates below 0.10 were used to indicate demographic independence^30^. The migration rates (*m*) for *G. coprotheses* grouped by geographic region showed that the highest gene flow occurred from the Middle region to the Northern region (*m* = 0.31; confidence interval (CI) = 0.30 to 0.32; Table 3). This is unsurprising as these birds are highly mobile and travel long distances to forage^14^ and the majority of these samples were collected from feeding sites. Interestingly, the rates of migration between the Western and Northern regions are low (*m* < 0.04), indicating that these two regions are demographically independent from each other. The results suggest that the Middle region may act as a source population for both the Western and Northern regions.

**Table 3:** Migration rates estimated using BayesAss for the 605 *Gyps coprotheres* individuals grouped by geographic region.

		Source	Middle	Northern
		Western		
Recipient	Western	**0.68 (0.67, 0.70)**	0.29 (0.26, 0.31)	0.03 (0.01, 0.05)
	Middle	0.00 (0.00, 0.01)	**0.98 (0.98, 0.98)**	0.02 (0.01, 0.02)
	Northern	0.00 (0.00, 0.01)	0.31 (0.30, 0.32)	**0.69 (0.68, 0.69)**

BayesAss estimates the fraction of migrants in each population from different sources (populations). The source populations are given in columns and recipient populations in rows. Bold values along diagonal are non-migrant proportions. The 95% confidence interval is provided in parentheses. Migration rates below 0.10 were used to indicate demographic independence^30^.

The migration rates between *G. coprotheses* collected at the six breeding colonies showed that the highest migration rates were observed out of the Collywobbles colony to all other colonies (*m* > 0.20; Table 4). Potberg, Msikaba, Umzimkulu, Skeerpoort and Kransberg colonies are all demographically independent from each other (*m* < 0.10).

**Table 4:** Migration rates estimated using BayesAss for only the 266 *Gyps coprotheres* collected at the six breeding colonies.

		Source	Collywobbles	Msikaba	Umzimkulu	Skeerpoort	Kransberg
		Potberg					
Recipient	Potberg	**0.68 (0.67, 0.69)**	0.23 (0.19, 0.27)	0.02 (0.00, 0.04)	0.03 (0.00, 0.06)	0.02 (0.00, 0.03)	0.02 (0.00, 0.04)
	Collywobbles	0.00 (0.00, 0.01)	**0.91 (0.83, 0.99)**	0.01 (0.00, 0.01)	0.07 (−0.01, 0.15)	0.01 (0.00, 0.01)	0.01 (0.00, 0.01)
	Msikaba	0.01 (0.00, 0.01)	0.24 (0.18, 0.29)	**0.69 (0.67, 0.70)**	0.06 (0.01, 0.11)	0.01 (0.00, 0.01)	0.01 (0.00, 0.01)
	Umzimkulu	0.01 (0.00, 0.01	0.27 (0.23, 0.30)	0.01 (0.00, 0.01)	**0.70 (0.67, 0.74)**	0.01 (0.00, 0.01)	0.01 (0.00, 0.01)
	Skeerpoort	0.01 (0.00, 0.01)	0.24 (0.21, 0.26)	0.01 (0.00, 0.01)	0.01 (0.00, 0.03)	**0.73 (0.71, 0.76)**	0.01 (0.00, 0.01)
	Kransberg	0.01 (0.00, 0.02)	0.26 (0.24, 0.29)	0.01 (0.00, 0.02)	0.02 (0.00, 0.03)	0.03 (0.01, 0.04)	**0.68 (0.67, 0.68)**

BayesAss estimates the fraction of migrants in each population from different sources (populations). The source populations are given in columns and recipient populations in rows. Bold values along diagonal are non-migrant proportions. The 95% confidence interval is provided in parentheses. Migration rates below 0.10 were used to indicate demographic independence^30^.

### Population bottleneck

Table 5 shows the results for the bottleneck analysis for all 605 *G. coprotheses* grouped by geographic region. The heterozygote excess method (Hx) for both mutation models showed no signs of recent bottleneck (p-value > 0.003). No significant deviation from the normal L-shaped distribution (Mode-shift) was observed in any of the regions. A similar result was observed when *G. coprotheses* from the six breeding colonies were analyzed (Table 5). The heterozygote excess method for both models showed no sign of a recent bottleneck (p-value > 0.05); neither did the Mode-shift test. Both the Wilcoxon test for heterozygote excess and the Mode-shift provide strong evidence that *G. coprotheres* populations, when considering both individuals grouped by region and only those individuals collected at the six breeding colonies, have not undergone a recent bottleneck.

**Table 5:** Bottleneck results (p-values) for the 605 *Gyps coprotheres* grouped by geographic region and for only the 266 *Gyps coprotheres* collected at the six breeding colonies.

		Wilcoxon test		Wilcoxon test		Sign test				Mode-shift
		One tailed for Hx		One tailed for Hd		SMM		TPM		
		SMM	TPM	SMM	TPM	Hd: Hx	p-value	Hd: Hx	p-value	
Region N = 605	Western	0.984	0.984	0.042	0.042	6:4	0.218	6:4	0.221	No
	Middle	1.000	1.000	**0.000**	**0.000**	13:0	**0.000**	13:0	**0.000**	No
	Northern	1.000	1.000	**0.000**	**0.000**	13:0	**0.000**	12:1	**0.000**	No
	All regions	1.000	1.000	**0.001**	**0.000**	13:0	**0.000**	12:1	**0.000**	No
Colony N = 266	Potberg	0.984	0.984	0.042	0.042	6:4	0.210	6:4	0.213	No
	Collywobbles	0.995	0.958	0.006	0.051	9:2	0.011	8:3	0.049	No
	Msikaba	0.991	0.920	0.012	0.097	7:3	0.066	7:3	0.075	No
	Umzimkulu	0.958	0.861	0.051	0.160	7:4	0.187	6:5	0.410	No
	Skeerpoort	0.884	0.813	0.138	0.216	7:3	0.081	6:4	0.230	No
	Kransberg	0.997	0.958	0.005	0.051	9:2	0.011	8:3	0.051	No
	All colonies	0.999	0.993	**0.000**	0.017	11:1	**0.001**	8:4	0.089	No

Two mutation models were used the stepwise mutation model (SMM) and the two-phase mutation model (TPM; using 90% SMM). Wilcoxon signed ranked tests for heterozygous excess (one tailed Hx) and heterozygous deficiency (one tailed Hd), a sign test and the Mode-shift test for bottleneck detection. Significant p-values are in bold and have been adjusted using the Bonferroni correction (p-value = 0.003).

Interestingly, two regions (Middle and Northern) showed significant heterozygosity deficiency (p-value < 0.003) for the Wilcoxon test under both mutation models. Additionally, the sign tests for the Middle region and Northern region indicated significant heterozygosity deficiency (p-value < 0.003) under both mutation models. Both regions significantly deviate from the expected ratio (1:1 for heterozygous deficiency to heterozygous excess) for non-bottlenecked, mutation equilibrium populations. When *G. coprotheres* was analyzed as a single population, a similar result was observed with significant heterozygosity deficiency (p-value < 0.003) for the Wilcoxon test under both mutation models. *G. coprotheres* population deviated from the expected ratio (1:1 for heterozygous deficiency to heterozygous excess) for non-bottlenecked, mutation equilibrium populations. None of the colonies deviated from the expected ratio for any of the tests. However, when all individuals collected from the six breeding colonies were observed as a single colony unit, significant deviation from the expected Wilcoxon test for heterozygous deficiency and sign test under SMM was observed. The heterozygous deficiency indicates that when the breeding colonies are considered as a single colony unit, they do not behave in mutation-drift equilibrium.

Effective population size (Ne) was estimated assuming both monogamous and random mating for *G. coprotheses*. The monogamous mating model estimated Ne = 409 individuals (confidence interval (CI): 318.5; 537.2) the ratio of effective population size to census population size (Ne/N)^31^ was 0.044. When the random mating model was selected Ne = 208 individuals (CI: 161.4; 274.6) Ne/N = 0.022. This is much lower than that reported number of *G. coprotheses* in recent census data (approximate 9400 individuals)^19^.

## Discussion

*G. coprotheres* has a relatively narrow distribution in comparison to other African vultures, which have continental scale distributions. As such this species provides a unique opportunity for us to intensively sample across the distribution of the species and thoroughly assess the genetic diversity of the species. This study represents the first comprehensive genetic assessment of the endangered *G. coprotheres* and provides important genetic baseline data for the conservation and management of the species.

Low levels of observed heterozygosity (mean Ho = 0.38) in *G. coprotheres* were recorded in this study. Genetic diversity estimates of *G. coprotheres* were also lower than that observed in the three other ecologically similar vulture species (*G. barbatus*, *N. monachus* and *G. africanus*). Although the sample sizes of the other three species were much smaller than that used for *G. coprotheres* the same genetic markers were amplified across all vulture species examined, allowing for direct comparison of levels of genetic diversity. This study found higher allelic diversity (number of alleles) in *G. coprotheres* probably as a result of differences in sample size, but, alarmingly *N. monachus* and *G. africanus* showed higher mean observed heterozygosity estimates. *N. monachus* revealed significantly higher levels of heterozygosity and low levels of inbreeding (F = −0.07). The genetic diversity values for *G. africanus* and *G. barbatus* estimated in this study are similar to the values reported in the literature for the Namibian *G. africanus* population^7^ and European *G. barbatus* populations^32^. This provides support for the results recovered in the present study not being biased by sample size or marker choice, but rather reflect the true levels of genetic variation present in populations of these African vultures. The lower than expected genetic diversity in *G. coprotheres* is of concern, given that both *N. monachus* and *G. africanus* are currently at higher extinction risk than *G. coprotheres. N. monachus* and *G. africanus* are currently listed as Critically Endangered by the IUCN^10^. The genetic diversity observed in *G. coprotheres* was lower than that observed in other threatened raptors such as the Spanish imperial eagle (*Aquila adalberti*)^3^; and the Eurasian black vulture (*Aegypius monachus*)^33^.

The Bayesian assignment tests indicated very shallow genetic structure across the range of the species. However, the pairwise F_ST_ values suggested genetic partitioning at a regional scale. Vultures from the two northern colonies (Skeerpoort and Kransberg) were genetically distinct from the other colonies. Given the geographic isolation and small size of the Potberg colony, we expected this colony to be the most effected by genetic drift. Although no marked allele frequency differences were observed in the STRUCTURE analysis, this colony did show the highest private allelic richness which is indicative of restricted gene flow. This is supported by the pairwise F_ST_ values in Supplementary Table 5 where Potberg is significantly dissimilar to the other five breeding colonies.

The shallow genetic structuring seen in this species was not unexpected; given that birds often show shallower genetic structuring than that of other vertebrate species^34–37^. The lack of genetic structuring in avian populations is attributed to high dispersal capabilities^38–40^. A similar pattern of low genetic structure was observed in Egyptian vulture (*Neophron percnopterus*)^41^ and griffon vulture (*G. fulvus*)^42^. In contrast, other wide-ranging vulture and raptor species such as the bearded vulture *G. barbatus*^43^, the Eurasian black vulture (*Aegypius monachus*)^33^; and the white-tailed eagle (*Haliaeetus albicilla*)^44^ have shown high levels of population differentiation.

The degree of natal philopatry (the likelihood that individuals breed at or near their place of origin) can influence the extent of genetic structuring in animal populations. Data from radio tracking and ringing studies have suggested that *G. coprotheres* exhibit natal philopatry^27,28^. Analysis of individuals collected from the six breeding colonies across South Africa did not support strong natal philopatry in this species, rather the genetic data suggests regional philopatry. The migration and pairwise F_ST_ results showed that the Potberg colony was demographically independent from all other colony pairs. The Skeerpoort and Kransberg colonies were also distinct, while the Collywobbles, Msikaba and Umzimkulu colonies seems to be operating as a single large regional unit.

*G. coprotheres* are listed as endangered by IUCN due to declines in overall population numbers^19^. Some recent surveys have however, reported increasing population number in some areas^45^. Populations that have experienced a recent bottleneck event are usually characterized by an excess of heterozygotes, as opposed to a population at mutation-drift equilibrium^46,47^. Over time however, inbreeding and genetic drift following a severe bottleneck event would lead to populations containing more homozygotes (heterozygote deficient). In the data generated in the present study, no signs of heterozygosity excess were observed in South African *G. coprotheres* populations. This could be because *G. coprotheres* may be currently experiencing a bottleneck and not enough generations have passed for the genetic signal of this event to be detected. In contrast, population genetic theory also suggests that *G. coprotheres* populations may have reduced genetic variation due to older bottleneck events^2,48^. This latter hypothesis was supported by results of Wilcoxon signed rank tests which found significant heterozygote deficiency in vultures from all three geographic regions and four colonies (Potberg, Collywobbles, Msikaba and Kransberg).

Identifying and prioritizing *G. coprotheres* populations, which have higher genetic variability, is an important step forward in conserving these birds. Genetically diverse populations could be valuable source populations for future translocation programmes. Six of the 24 localities showed elevated heterozygosity (F < 0; (Commando Drift and Elliot in Eastern Cape; Natal Midlands in KwaZulu-Natal; Soetdoring in Freestate; Blouberg in Limpopo; and Mala Mala in Mpumalanga), whereas all other localities showed reduced heterozygosity. The isolated Potberg colony in the Western Cape had the lowest genetic variation of all the breeding colonies. This is not unexpected given the isolated nature of this colony. Careful management and monitoring of this colony is needed to avoid the detrimental effects of inbreeding and genetic drift. Given the shallow genetic structuring observed across the range of the species and the high levels of gene flow among breeding colonies, the present study suggests that the entire South African *G. coprotheres* population be managed as a single management unit.

The results from this study showed low levels of genetic diversity and variability in South African *G. coprotheres* populations. This, together with the rapid decline of this species^9,21^, highlights the need for conservation strategies to include the maintenance of genetic diversity as a population management tool. Reductions in genetic diversity can have serious implications on the evolutionary potential of a species^49^. The present study highlights the utility of microsatellite markers for the assessment and monitoring of genetic diversity in an African vulture species. The data produced in this study can be used as a baseline for future genetic monitoring and species recovery programmes.

## Material and Methods

### Sampling

A total of 605 *G. coprotheres* from 24 localities, across the South African distribution of the species, were sampled for this study (Supplementary Table 8). Based on sample localities, samples were grouped into three geographic regions: Western (n = 18), Middle (n = 462) and Northern (n = 125). In addition to the regional grouping, the 266 samples collected from six breeding colonies (Fig. 1) were analysed separately. South African representatives of *G. barbatus* (n = 54), *N. monachus* (n = 54), and *G. africanus* (n = 68) were also included and genotyped using the same microsatellite loci, to allow for direct comparison of genetic diversity values.

Samples consisted of feather samples, collected opportunistically from feeding sites, sites of electrocutions, poisoning events and below nests at six main breeding colonies^18^. Blood samples were also collected when vultures were captured and fitted with global positioning system/global system for mobile transmitters^14^. Bloods were stored on Whatman FTA® Elute cards (Sanford, USA). Archival museum samples (dried skin snips) were sourced from local South African museums (Supplementary Table 8). Ethical approval was obtained for this study from the University of KwaZulu-Natal Animal Ethics subcommittee (Reference number: 045/15/Animal) and all experiments were performed in accordance with relevant guidelines and regulations.

### DNA extraction

The NucleoSpin® Tissue kit (Macherey-Nagel, Germany) was used for all DNA extractions. Extractions protocols were modified slightly for feather and archival samples to improve DNA yield. Modifications included: incubation of the sample and proteinase K for 48 hours in a shaking water bath (56 °C), the lysate was incubated in B3 buffer for 45 minutes (70 °C), the final volume of pre-warmed Buffer BE was decreased to 80 μl and the samples were incubated at 70 °C for 20 minutes followed by centrifuging and then reapplication of the solution onto the membrane and a final incubation at 70 °C for an additional five minutes followed by a final centrifuging step.

### Microsatellite amplification

Thirteen microsatellite loci were chosen from a suite of markers used in previous studies of *G. fulvus*^50^ and *G. barbatus*^51^ (Supplementary Table 9). The 13 loci were amplified in six multiplex reactions (Supplementary Table 9) using the KAPA2G^TM^ Fast Multiplex PCR kit (KAPA Biosystems). The 10 μl reactions consisted of ~2–30 ng template DNA, 5 μl KAPA2G Fast Multiplex mix, 0.2 μM of each primer, 0.1 μl of 1 mg/ml bovine serum albumin (BSA) and purified water. In reactions performed using DNA extracted from feather and archival samples, template DNA was increased to ~20–200 ng to improve amplification success. The cycling parameters for all loci followed the standard KAPA2G^TM^ Fast Multiplex PCR kit protocol except for multiplex 1, where the annealing temperature was reduced to 58 °C. All amplified products were sent to the Central Analytical Facility at Stellenbosch University, South Africa for fragment analysis. The software GeneMarker v2.4.0 (Soft Genetics) was used for genotype scoring. To ensure genotyping consistency, all archival samples were re-amplified, and each locus was genotyped multiple times (up to five times). In addition, 20% of all feather, muscle and blood samples were re-amplified multiple times (up to five times) to verify the reliability of the data.

### Data analysis

#### Assessing genetic variation

To ensure that duplicated genotypes were not included when feathers were collected, identity analysis was performed in Cervus v3.0.7^52^. Cervus was also used to estimate polymorphic information content (PIC) for each locus. Null allele frequencies for each locus were estimated in FreeNA^53^ using the expectation maximization (EM) algorithm^54^. Because null alleles can bias population structure analysis^55^, uncorrected global F_ST_ were compared to F_ST_ values corrected using the excluding null alleles (ENA) method^53^ using a paired t-test. Linkage disequilibrium was tested using Genepop v4.2^56^. Deviations from Hardy-Weinberg equilibrium were estimated in GenAlEx v6.502^57^. The effective population size (Ne) was estimated in NeEstimator v2.01^58^ implementing both random and monogamous mating.

Genetic diversity estimates (number of alleles, observed heterozygosity, unbiased expected heterozygosity and inbreeding coefficient) were calculated in GenAlEx. Tests for deviation from Hardy-Weinberg equilibrium were performed in Genepop. Allelic richness was estimated in FSTAT v2.9.3.2^59^. The observed number of alleles in a population is dependent on sample size and given the opportunistic nature of sampling used in this study sample size varied across localities. Consequently, allelic richness was calculated using a rarefaction index estimated in FSTAT and the number of private alleles within populations were estimated using the rarefaction method implemented in HP-RARE v1.0 software^60^. All multiple comparisons were adjusted using the Bonferroni correction.

### Population structure

Bayesian assignment tests were performed in STRUCTURE v2.3.4.^61^. For each analysis ten independent runs were performed consisting of 100000 Markov chain Monte Carlo (MCMC) replicates with a burn-in of 10000 and the proposed number of genetic clusters (K) ranging from one to ten. The admixture ancestry model with correlated allele frequencies was selected for all runs. Sampling locality information was incorporated using the LOCPRIOR model. STRUCTURE Harvester^62^ was used to estimate the most probable number of genetic clusters^63^. Membership probabilities (Q-values) for each individual and for each genetic cluster were estimated using ClumpAK^64^. Bar plots from STRUCTURE runs were created in Pophelper^65^.

The correct identification of the number of genetic clusters can be challenging if there is unbalanced sampling or complex phylogeographic structure i.e. when there are unequal genetic distances among subpopulations or unbalanced sample sizes^66^. For this reason, Principle Coordinate Analysis (PCoA) was also performed in GenAlEx using pairwise codominant genotypic genetic distance. PCoA does not rely on a model but rather multidimensional scaling to visualize similarity in a dataset.

Analysis of molecular variance (AMOVA) was performed in GenAlEx. Three AMOVA’s were conducted, one grouping individuals by sampling locality, a second grouping individuals by geographic region. A third AMOVA was conducted only on individuals collected from the six breeding colonies. Pairwise F_ST_ values were estimated in FSTAT. Mantel tests^67^ were performed in GenAlEx, to test for correlation between geographic distance and genetic distance.

### Population connectivity

Migration rates (gene flow) between the three geographic regions and the six breeding colonies were estimated in BayesAss v1.3^68^. Two analyses were performed consisting of 10000000 iterations, with a burn-in of 1000000 iterations and a sampling frequency of 90000 iterations. Multiple runs were performed with varying run lengths to ensure MCMC chain convergence. The delta values for each parameter were adjusted to achieve a 20–60% acceptance rate^68^. In analysis to estimate gene flow among the three geographic regions, the final delta values were delta allele frequency = 0.30, delta migration rate = 0.10 and delta inbreeding coefficient = 0.50. In analysis to estimate gene flow among the six breeding colonies, the final delta values were delta allele frequency = 0.50, delta migration rate = 0.10 and delta inbreeding coefficient = 0.70. For both analyses, migration rates below 0.10 were used to indicate demographic independent populations^30^.

### Population bottleneck

BOTTLENECK v1.2.02^69^ was used to test for evidence of heterozygosity excess (Hx)^5,47^ in *G. coprotheres* populations. Two analyses were performed, one grouping individuals by geographic region, and a second only including individuals collected at the six breeding colonies. Each analysis ran two mutation models. The conservative stepwise mutation model (SMM) and the two-phase model (TPM) with 90% stepwise mutation^70,71^ and a variance of 12^69^ to include the observed range of multistep mutations in natural populations^72^. Two statistical tests (Wilcoxon sign-rank test and the sign test) as well as a qualitative test (Mode-shift test), were performed. The Wilcoxon sign-rank test is effective in detecting recent declines in effective population size (*Ne*) and assumes that populations that have recently undergone a decline in effective population size will have a higher level of heterozygosity compared to a population at mutation-drift equilibrium^5^. The sign test was used to identify the number of microsatellite loci that have either heterozygosity excess (Hx) or heterozygosity deficiency (Hd). Changes in heterozygosity (excess or deficit) can occur after a recent change in the effective population size or if heterozygotes have a selective advantage or disadvantage^69^. In populations experiencing a bottleneck event, alleles are usually lost faster than heterozygosity, thus producing a heterozygote excess^47^. The Mode-shift is a qualitative indicator that can differentiate between bottlenecked and stable (non-bottlenecked) populations. This test can detect the genetic changes caused by a population decline within a few dozen generations^73^. Therefore, only recent bottleneck events will be identified using this method.

## Supplementary information

Supplementary Table

## Data Availability

The data analysed in this study will be made available as Supplementary information upon publication.
